# Beyond the undergraduate experience: Pre-arrival expectations of postgraduate taught students at a UK university

**DOI:** 10.1371/journal.pone.0358341

**Published:** 2026-09-15

**Authors:** Angela Hibbs, Rick Hayman, Danielle Grenade, Remco Polman

**Affiliations:** 1 Department of Sport, Exercise and Rehabilitation, Northumbria University, Newcastle upon Tyne, United Kingdom; 2 School of Exercise and Nutrition Sciences, Queensland University of Technology, Brisbane, Australia; 3 Department of Health and Physical Education, The Education University of Hong Kong, Hong Kong, China; Indira School of Business Studies, INDIA

## Abstract

Postgraduate taught (PGT) students represent a rapidly growing yet under‑researched population in higher education, often characterised as the “forgotten sector”. This study examined pre-arrival confidence and importance ratings among 1332 first-year PGT students (68% response rate) across twelve academic disciplines at a UK university, considering discipline, gender, international student status, and socioeconomic background. Using a validated Pre-Arrival Survey measuring learning, community, employability, and health/well-being domains, we employed confirmatory factor analysis, MANOVA, and ANCOVA to examine group differences. PGT students rated importance higher than confidence across all domains, though this gap was narrower than in undergraduate populations. Community confidence ranked lowest across 11 of 12 disciplines, highlighting persistent integration challenges. International students demonstrated significantly higher confidence and importance ratings across nearly all domains. Gender differences observed at undergraduate level were largely absent, with males showing higher scores only for two of the eight factors; community confidence and employability importance. Socioeconomic indicators showed no significant effects. Computer science students reported highest confidence while healthcare/nursing sciences reported lowest scores. Findings suggest PGT students arrive with more calibrated expectations than undergraduates but require differentiated transition support, particularly for community building. The validated Pre-Arrival Survey provides institutions with a tool for understanding incoming PGT cohorts and adapting provision to support this increasingly diverse student population.

## Introduction

The transition to postgraduate taught (PGT) education represents a critical yet under-researched phase in the higher education (HE) journey. Whilst transitions from school to undergraduate (UG) study have received considerable scholarly attention [1,2]. the experiences and expectations of students entering PGT programmes have been characterised as belonging to the “forgotten sector” of HE [3]. PGT students are often presumed to be educational experts who require minimal support, having already navigated UG study successfully [3]. However, research increasingly challenges this assumption, revealing that transitions to PG study can be characterised by anxiety, self-doubt and disorientation, particularly regarding unclear academic expectations and limited opportunities for integration and belonging [3,4]. PGT programmes present unique challenges: they are typically intensive (i.e., block teaching days), compressed into 12 months and require students to demonstrate higher-level critical thinking and independent scholarship whilst simultaneously managing career development aspirations (i.e., paid employment, work experience) [4]. Furthermore, the PGT cohort is highly heterogeneous, encompassing mature students returning to education, recent UGs pursuing further qualifications, career changers and a substantial proportion of international students seeking enhanced employability prospects [5]. Within some degree pathways heterogeneity may vary by discipline due to aspects such as entry requirements or type of institution (Post-92 versus traditional ‘red brick’ universities). Traditionally, Post-92 universities typically recruit more UK based students and those from widening participation backgrounds compared to traditional Russell Group universities.

Pre-arrival expectations play a crucial role in shaping educational transitions, influencing engagement, persistence, and ultimate success in HE [6,7]. Students who arrive with realistic expectations demonstrate better adjustment and well-being than those whose expectations diverge significantly from reality [8,9]. However, whilst pre-arrival expectations of UG students have been examined [10], equivalent research focusing specifically on PGT cohorts remains scarce [3].

Hibbs et al. [11] examined pre-arrival confidence and importance ratings among first-year UG students across twelve academic disciplines, revealing significant variations by programme of study, gender, and international student status. Psychology students reported significantly lower pre-arrival scores compared to other disciplines, female students rated learning, community, and health and well-being importance significantly higher than males, and international students demonstrated higher confidence and importance ratings across all domains. These findings challenged assumptions of uniform student experiences and highlighted the necessity for tailored institutional support acknowledging disciplinary and demographic differences.

Building upon this UG foundation, the present study extends these findings to the PG taught population. Several factors suggest PGT expectations may differ substantially from undergraduate expectations. Firstly, PGT students have already experienced HE, potentially arriving with more realistic or refined expectations based on prior experience [12]. Secondly, many PGT students pursue postgraduate study for career advancement, suggesting employability confidence and importance may be similar [13]. Thirdly, the compressed, intensive nature of PGT study may heighten anxiety regarding academic preparedness, particularly for students transitioning from different disciplines or returning to education after time in employment. Finally, the high proportion of international PGT students introduces additional complexity regarding expectations shaped by diverse educational backgrounds and cultural contexts [14].

Gender differences in academic confidence and perceived importance, well-documented at UG level [11], warrant examination within PGT contexts. Research consistently demonstrates that UG female students prioritise intrinsic learning objectives and holistic well-being, whilst male students may emphasise extrinsic goals such as financial returns and career advancement [15,16]. At PGT level, these differences may be amplified depending on life stage, prior work experience, and programme characteristics. Understanding gender differences in PGT expectations is essential given the potential for expectation-reality mismatches to contribute to attrition or dissatisfaction and the persistent under-representation of women in certain postgraduate fields (i.e., STEM disciplines). Delaney and Devereux [17] found that female students were 20% less likely to enrol on a STEM PGT degree than male students.

International students make up a substantial proportion of the UK PGT population, with PGT international enrolments increasing by approximately 43% between 2021 and 2022 [18]. These students represent a highly self-selected population who have overcome considerable barriers including visa processes, financial investments, and geographical relocation [19]. Employability represents a primary driver for international PGT study, with research indicating that future career impact ranks as the foremost consideration influencing institutional choice [20]. However, international PGT students simultaneously face distinct challenges including language barriers, unfamiliar pedagogical practices, social isolation and limited understanding of UK labour market expectations [21,22] all of which may have a significant impact on their academic, community and employability confidence.

Socioeconomic background influences educational aspirations and self-confidence through multiple pathways [23,24]. First-generation university students and those from disadvantaged backgrounds often navigate HE without familial experience or established social capital [25]. At PGT level, these challenges may be compounded by financial pressures, as PGT study typically lacks the funding support available for UG education [5]. However, some research suggests that socioeconomic differences in confidence may diminish once students commit to PG study, possibly indicating that PGT cohorts represent particularly resilient subgroups who have demonstrated capacity to overcome structural barriers [26]. However, currently there is no published research on this issue.

The present study examines PGT student pre-arrival confidence and perceived importance across twelve academic disciplines at a UK HE institution, considering the influence of gender, international student status and socioeconomic indicators. By employing the same validated pre-arrival survey instrument used by Hibbs et al. [11] in a preceding UG study at the same UK HE institution, direct comparisons can be drawn regarding whether patterns observed at UG persist at PG level or whether the PG cohort demonstrates distinct characteristics. Findings will inform sector-wide transition programmes and the design and delivery of teaching and learning materials specifically for PGT students and contribute to theoretical understanding of how student expectations vary across educational levels, academic disciplines, and demographic characteristics.

## Methods

### Participants

Of 1964 eligible participants (newly arriving students embarking on either a masters level degree programme or a postgraduate certificate) 1332 (68%) completed the pre-arrival survey, representing 744 (56%) male, 588 (44%) female first-year postgraduate students. Both full-time and part-time students on the relevant postgraduate degree programmes were invited to take part. Out of the 520 postgraduate degree taught programmes at the UK higher education institution 370 programmes were represented in the sample. These programmes of study were grouped into twelve disciplines (number of programmes included in the sample in brackets); business (76), law (47), architecture and built environment (21), communities and education (55), computer science (61), design arts and creative industries (25), engineering physics and mathematics (3), geography and natural sciences (6), healthcare and nursing sciences (58), humanities and social sciences (8), psychology (3) and sport exercise and rehabilitation (5). Survey responses per gender and discipline can be seen in Table 1. The heterogeneity of our sample reflects the wider UK PGT sector, which is characterised by diverse student pathways, including mature returners, recent graduates, career changers and a substantial international population, consistent with national patterns describing PGT cohorts as markedly non-uniform.

**Table 1:** Respondent demographic per discipline of study.

Discipline	Total	Gender		UK resident		Free school meals			First generation student		
		Female	Male	Yes	No	Yes	No	Other	Yes	No	Other
**Business**	497	204	293	25	472	14	285	198	9	5	483
**Law**	57	34	23	13	44	2	36	19	6	6	45
**Architecture and Built Environment**	140	47	93	12	128	7	82	51	4	5	131
**Communities and Education**	118	88	30	67	51	15	63	40	24	35	59
**Computer Science**	283	65	218	18	265	9	166	108	4	5	274
**Design, Arts and Creative Industries**	41	31	10	13	28	4	19	18	9	5	27
**Engineering, Physics and Mathematics**	30	3	27	–	30	1	18	11	–	–	30
**Geography and Natural Sciences**	44	30	14	9	35	2	31	11	4	4	36
**Healthcare and Nursing Sciences**	29	23	6	29	–	1	14	14	11	12	6
**Humanities and Social Sciences**	40	28	12	20	20	6	21	13	12	9	19
**Psychology**	39	27	12	26	13	3	24	12	12	11	16
**Sport, Exercise and Rehabilitation**	14	8	6	8	6	0	9	5	5	3	6
**Total**	1332	588	744	240	1092	64	768	500	100	100	1132

### Procedure

Between the 1^st^ September 2025 till 19^th^ September 2025, all newly enrolled and pre-enrolled first-year PGT students at the participating institution were invited to take part in the study. Following the granting of institutional ethical clearance, relevant student information, including name, gender, programme of study, and personal email address, was obtained from the institution’s academic registry post-clearing database. Survey distribution was facilitated using Explorance Blue (www.explorance.com), ensuring efficient dissemination and collection of responses. Each student received a unique survey link via an institution-branded, personalised email. The survey commenced with an overview outlining the study’s aims, objectives, and procedures. Participants provided informed consent and were notified of their right to withdraw at any time without explanation; anonymity was maintained through the assignment of unique student identifiers. Four waves of the survey were distributed: the first in week 1 to all enrolled students, and subsequent rounds in weeks 2, 3, and 4 to all newly enrolled first-year students. The survey period concluded on the Friday preceding welcome week in mid-September 2025. Students who did not respond to the initial invitation received a reminder email with an additional opportunity to participate one week later.

### Design and analysis

The survey structure was originally developed by Lawson and has been previously utilised to assess pre-arrival confidence levels among further education students [10]. The Pre-Arrival Survey (PAS) primarily consists of closed questions, facilitating efficient and straightforward completion through a combination of yes/no and Likert scale items. Participants provided responses relating to four factors: learning (L), community (Co), employability (E), and health and well-being (H), within two domains: confidence (C) (“I am confident with...”) and importance (I) (“I think it is important to...”). The survey included 45 statements, each corresponding to one of eight factor-domain combinations (LC: 7 items, LI: 5 items, CoC: 6 items, CoI: 4 items, EC: 7 items, EI: 7 items, HC: 6 items, HI: 3 items). Respondents rated their perceived level of confidence or importance for each statement on a four-point Likert scale ranging from strongly agree (4) to strongly disagree (1), where 4 indicated the highest level of confidence or importance.

We calculated the total PAS score, the two domain scores and the factor scores in each domain. The total PAS score represents an overall indicator of how prepared and motivated students feel as they enter their PGT programme. In practical terms, the total score provides a single metric reflecting the extent to which students believe they are ready to meet the academic, social and professional demands of PGT study. Higher scores indicate a stronger perceived readiness and value placed on the 4 factors in each domain whereas lower scores indicate that students may require additional transition support. The total score, therefore, provides a broad screening indicator to identify incoming cohorts or subgroups who could benefit from early intervention, targeted induction activities, or enhanced academic and well-being support. The importance domain reflects the extent to which students perceive specific learning, community, employability and well-being behaviours or outcomes as important for their forthcoming PG experience and success. It is associated with their prioritisation of these activities. Higher importance scores indicate the areas students consider most important for their academic and personal success as they prepare to begin their programme of study. Practically, it provides insight into their expectations of institutional support and the aspects of the transition they consider most important. Finally, the confidence domain reflects the students self-perceived readiness to which they feel capable of engaging with learning, community, employability and health and well-being activities. Higher confidence scores indicate stronger perceived readiness prior to arrival. Practically, this provides insight into the areas where students believe they already possess the required skills or understanding and the factors they may require additional support. Demographic questions gathered socio-economic status data, including whether respondents were residents of the UK, had received free school meals during compulsory education, previous qualification level, and first-generation student status. A copy of the survey is available upon request from the primary author.

### Analysis strategy

Prior to analysis we examined the data for normality (Levene) and outliers to ensure compliance. Following this we began by evaluating the psychometric characteristics of the PAS, conducting separate confirmatory factor analyses (CFA) for both the Confidence and Importance domains. These analyses were performed using JAMOVI with the maximum likelihood estimation method, drawing on several fit indices: (a) the Chi square statistic, where P > .05 suggests an acceptable model fit, though this measure is sensitive to sample size (Anderson & Gerbing, 1998); (b) the Goodness of Fit Index (GFI), Comparative Fit Index (CFI) and Tucker Lewis Index (TLI), with values above.95 denoting a good fit and those over.90 considered acceptable (Hu & Bentler, 1999); and (c) the Root Mean Square Error of Approximation (RMSEA) and Standard Root Mean Square Residual (SRMR), with scores ≤ .06 reflecting a good fit and those > .6 and ≤.08 regarded as acceptable (Browne & Cudeck, 1992). Where the model fit proved inadequate, modifications were made based on modification indices, and error terms were only correlated within the same factor due to theoretical similarities among items (Byrne, 1994). To assess reliability, Cronbach’s alpha was calculated at both the domain and factor levels.

Next, descriptive statistics (mean and standard deviation) were obtained for the PAS as a whole, the two domains (Confidence and Importance) and the 8 factors (LC, LI, CoC, CoI, EC, EI, HC, HI) for gender and discipline of study. The responses per domain and factor were ranked by discipline of study with highest scores ranking first. Following this, we examined whether socio-economic status (free school meals & first in generation) and being international or domestic student influenced the PAS total (independent t-test), domain (Multivariate Analysis of Variance (MANOVA)) or factor (MANOVA) score. The number of international students was not evenly distributed across disciplines. As such, in the instance of an effect for this variable we controlled for this in subsequent analysis.

Separate univariate analysis of covariance (ANCOVA) was conducted for gender (n = 2) and discipline of study (n = 12) as independent variables and being an international student (n = 2) as a covariate for total PAS score. Multivariate analysis of co-variance (MANCOVA) was conducted separately for gender and discipline of study as the independent variables and being an international student as the covariate for the two domains (Confidence and Importance) and the eight factors (LC, LI, CoC, CoI, EC, EI, HC, HI). No interactions between gender and discipline of study were explored due to low participant numbers in some cohorts. Sidak post-hoc comparisons were conducted for a significant main effect for discipline of study. Effect sizes were calculated using partial eta squared (η_p_^2^) with.01- < .6 = small;.06 – < .14 = medium; and >.14 = large (Cohen, 1988). Significance level was set at P < .05. All data were analysed in SPSS (v. 28).

## Results

### Confirmatory factor analysis

An acceptable fit was obtained following cross-correlating the error terms of HC items 4–5 and EC 3–4 for Confidence and EI items 6–7 for Importance (see Table 2). The Chi Square was significant for both domains likely due to the relatively large sample size. The other fit indices indicated a good (GFI; CFI; RMSEA; SRMR) or adequate (CFI; TLI) fit. Reliability analysis showed an acceptable (EI α = .78), good (LC α = .84; LI α = .86; CoC α = .88; CoI α = .86; HC α = .85; HI α = .84) or excellent (EC α = .93) reliability for the confidence and importance domains and factors (see Table 2). These findings indicate acceptable reliability and factorial structure for the PAS.

**Table 2:** Fit indicators for the confidence and importance domains of the PAS. GFI = Goodness of Fit Index, CFI = Comparative Fit Index; TLI = Tucker Lewis Index; RMSEA = Root Mean Square Error of Approximation; SRMR = Standard Root Mean Square Residual).

Fit indicator	Confidence	Importance
**Chi Square**	1588; P < .001	732; P < .001
**GFI**	.99	.99
**CFI**	.95	.93
**TLI**	.93	.94
**RMSEA**	.058	.055
**SRMR**	.043	.051
**Cronbach α**	.94	.88

### Socioeconomic and international/domestic analysis

For school meals there was no effect for total PAS score (see Table 3). The analysis for the two domains showed that those receiving a school meal scored significantly lower on confidence but not importance. This was due to significant differences for CoC, CoI and HC with those receiving school meals scoring significantly lower than those who did not although the effect size was small. For parental education there were no significant differences in total PAS, domain or factor scores for those who were first in family to go to university versus those that were not (see Table 3). The international students scored significantly higher compared to the domestic students on the total PAS, both domain and 7 of the 8 factor scores (see Table 3). Because of this difference we controlled for this variable in the subsequent analysis.

**Table 3:** Socioeconomic and international student status effect from the follow-up ANOVA per total PAS, domain and factor scores (LC = Learning Confidence; LI = Learning Importance; CoC = Community Confidence; CoI = Community Importance; EC = Employment Confidence; EI = Employment Importance; HC = Health and Wellbeing Confidence; HI = Health and Wellbeing Importance, * Indicates a significant difference).

	Free School meals			First generation student			International student status		
	t	P	Cohens d	T	P	Cohens d	t	P	Cohens d
**Total PAS**	2.01	.05	.26	1.15	.25	.16	12.97	<.001*	.92
	F(1,831)		η_p_^2^	F(1,936)		η_p_^2^	F(1,1331)		η_p_^2^
**Domain**	Wilks’ λ = .95; P = .02; η_p_^2^ < .01*			Wilks’ λ = .82; P = .14; η_p_^2^ = .02			Wilks’ λ = .95; P < .001; η_p_^2^ = .12*		
**Confidence**	6.41	.01*	.01	3.18	.08	.01	185.68	<.001*	.12
**Importance**	.89	.35	<.01	.008	.93	<.01	101.27	<.001*	.07
**Factor**	Wilks’ λ = .85; P = < .01; η_p_^2^ = .03*			Wilks’ λ = .72; P = .06; η_p_^2^ < .07			Wilks’ λ = .94; P < .001; η_p_^2^ < .21*		
**LC**	.90	.34	<.01	1.55	.22	<.01	53.67	<.001*	.04
**LI**	.05	.83	<.01	.49	.48	<.01	23.29	<.001*	.02
**CoC**	11.45	<.01*	.01	5.35	.02*	.03	285.05	<.001*	.18
**CoI**	9.94	<.01*	.01	2.62	.11	.01	199.08	<.001*	.13
**EC**	2.10	.15	<.01	.02	.90	<.01	63.83	<.001*	.05
**EI**	.16	.69	<.01	.37	.54	<.01	73.99	<.001*	.05
**HC**	4.26	.04*	.01	2.13	.15	.01	100.67	<.001*	.07
**HI**	.39	.53	<.01	2.72	.10	.01	1.70	.19	<.01

### Pre-arrival overall score

The ANCOVA for the PAS overall score was significant for discipline of study (F(11,1332) = 1.81; P < .05; η_p_^2^ = .02) but no significant gender effect were observed (F(1,1332) = 2.64; P = .10; η_p_^2^ < .01). While the ANCOVA identified a significant discipline of study difference, Sidak post-hoc comparisons failed to identify any significant differences between discipline of study.

### Domain and pre-arrival factors

For the domain scores, the MANCOVA showed a significant discipline of study (Wilks’ λ = .91; P < .001; η_p_^2^ = .07) but no gender (Wilks’ λ = .38; P = .25; η_p_^2^ < .01) main effect. Follow up ANCOVA showed a significant discipline of study effect on pre-arrival confidence (F(11,1332) = 1.63; P = .01; η_p_^2^ = .02) but not importance (F(11,1332) = 1.63; P = .09; η_p_^2^ = .01). Sidac post-hoc comparisons showed computer science students reported higher confidence scores than geography students.

For pre-arrival factor scores, the MANCOVA showed a significant discipline of study (Wilks’ λ = .90; P < .001; η_p_^2^ = .02) and gender (Wilks’ λ = .86; P < .01; η_p_^2^ = .02) main effect. Follow-up ANCOVA results are presented in Table 4. Sidak post-hoc comparisons for discipline of study showed that for LC computer science,humanities and social sciences and architecture and built environment reported significantly higher learning confidence than students in healthcare and nursing sciences. For EC, computer science students reported higher scores than business and psychology students. For CI, students studying healthcare and nursing sciences reported lower scores than 10 of the other disciplines of study (all except humanities and social sciences). For CC, while the MANOVA detected a significant discipline of study difference, post-hoc comparisons failed to identify any significant differences between disciplines of study (see Table 5). Sidak post-hoc comparisons for gender showed that for CoC and EI male students reported higher pre-arrival scores than female students.

**Table 4:** Pre-arrival factor and domain results of the follow-up ANCOVA for gender and discipline of study main effect (LC = Learning Confidence; LI = Learning Importance; CoC = Community Confidence; CoI = Community Importance; EC = Employment Confidence; EI = Employment Importance; HC = Health and Wellbeing Confidence; HI = Health and Wellbeing Importance; C = Confidence domain; I = Importance domain).* Indicates a significant difference.

	Gender			Discipline of Study		
	F(1,1332)	P	η_p_^2^	F(11,1332)	P	η_p_^2^
LC	.66	.43	<.01	2.55	<.01*	.02
LI	1.08	.30	<.01	1.21	.27	.01
CoC	7.55	<.01*	<.01	2.80	.001*	.03
CoI	.18	.67	<.01	4.26	<.001*	.04
EC	1.47	.23	<.01	2.25	.01*	.02
EI	4.42	<.04*	<.01	1.27	.24	.01
HC	.06	.80	<.01	1.53	.11	.01
HI	3.68	.06	<.01	.74	.70	<.01
C	2.72	.10	.11	2.26	.01*	.02
I	1.77	.18	.06	1.63	.09	.01

**Table 5:** Mean and standard deviation for the 8 factors per discipline of study by gender (LC = Learning Confidence; LI = Learning Importance; CoC = Community Confidence; CoI = Community Importance; EC = Employment Confidence; EI = Employment Importance; HC = Health and Wellbeing Confidence; HI = Health and Wellbeing Importance; M = Male; F = Female).

Discipline of Study	Gender	LC	LI	CoC	CoI	EC	EI	HC	HI
**Business**	M	3.46 0.39	3.64 0.39	3.45 0.44	3.48 0.46	3.46 0.49	3.38 0.42	3.50 0.41	3.37 0.53
	F	3.41 0.41	3.70 0.38	3.35 0.48	3.50 0.44	3.36 0.57	3.31 0.47	3.47 0.44	3.28 0.61
**Law**	M	3.37 0.49	3.52 0.49	3.08 0.77	3.28 0.58	3.29 0.59	3.30 0.46	3.35 0.51	3.45 0.48
	F	3.49 0.44	3.64 0.40	3.21 0.62	3.38 0.61	3.52 0.59	3.35 0.41	3.55 0.43	3.40 0.62
**Architecture & Built Environment**	M	3.48 0.38	3.67 0.40	3.37 0.50	3.47 0.42	3.44 0.56	3.33 0.42	3.48 0.41	3.37 0.52
	F	3.42 0.34	3.67 0.39	3.40 0.37	3.56 0.42	3.49 0.45	3.41 0.34	3.57 0.42	3.46 0.55
**Communities & Education**	M	3.32 0.44	3.67 0.42	3.05 0.74	3.25 0.82	3.32 0.67	3.21 0.67	3.41 0.50	3.51 0.52
	F	3.31 0.43	3.62 0.41	3.05 0.51	3.27 0.60	3.35 0.54	3.16 0.54	3.35 0.49	3.34 0.55
**Computer Science**	M	3.47 0.38	3.70 0.37	3.44 0.46	3.56 0.44	3.52 0.51	3.41 0.40	3.51 0.41	3.38 0.57
	F	3.55 0.36	3.76 0.36	3.49 0.47	3.61 0.43	3.63 0.50	3.40 0.38	3.62 0.39	3.41 0.59
**Design, Arts & Creative Industries**	M	3.56 0.31	3.88 0.17	3.50 0.59	3.70 0.42	3.59 0.53	3.54 0.42	3.48 0.62	3.40 0.58
	F	3.37 0.38	3.59 0.40	2.96 0.52	3.36 0.42	3.25 0.60	3.22 0.40	3.30 0.48	3.21 0.63
**Engineering, Physics and Mathematics**	M	3.41 0.42	3.69 0.38	3.41 0.49	3.56 0.40	3.47 0.58	3.49 0.42	3.60 0.31	3.46 0.50
	F	3.29 0.29	3.73 0.31	3.06 0.34	3.08 0.14	3.09 0.50	3.42 0.29	3.33 0.86	3.00 0.67
**Geography & Natural Sciences**	M	3.37 0.41	3.75 0.32	3.38 0.44	3.45 0.38	3.25 0.55	3.39 0.35	3.41 0.49	3.41 0.62
	F	3.31 0.35	3.67 0.39	3.09 0.61	3.37 0.43	3.24 0.57	3.30 0.42	3.27 0.42	3.40 0.53
**Healthcare & Nursing Sciences**	M	3.00 0.46	3.70 0.32	2.81 0.66	3.04 0.68	3.52 0.47	3.26 0.89	3.56 0.50	3.67 0.52
	F	3.04 0.46	3.37 0.41	2.69 0.62	2.51 0.74	3.10 0.51	2.87 0.63	3.20 0.54	3.32 0.62
**Humanities & Social Sciences**	M	3.51 0.47	3.85 0.28	3.21 0.56	3.46 0.46	3.38 0.47	3.42 0.41	3.56 0.59	3.64 0.56
	F	3.46 0.41	3.67 0.43	2.98 0.59	3.09 0.63	3.26 0.58	3.25 0.35	3.38 0.48	3.38 0.62
**Psychology**	M	3.07 0.43	3.60 0.43	2.93 0.66	3.29 0.53	3.05 0.40	3.17 0.37	3.43 0.52	3.53 0.72
	F	3.24 0.28	3.72 0.39	2.98 0.53	3.28 0.42	3.07 0.51	3.25 0.33	3.22 0.54	3.23 0.65
**Sport, Exercise & Rehabilitation**	M	3.14 0.64	3.50 0.45	3.19 0.51	3.25 0.47	3.33 0.44	3.14 0.34	3.47 0.27	3.50 0.59
	F	3.47 0.45	3.73 0.45	3.50 0.38	3.82 0.35	3.55 0.55	3.39 0.35	3.67 0.35	3.29 0.70
**Total**	M	3.44 0.40	3.67 0.45	3.39 0.51	3.49 0.48	3.45 0.52	3.38 0.43	3.49 0.42	3.40 0.54
	F	3.39 0.41	3.67 0.39	3.22 0.54	3.40 0.54	3.37 0.56	3.28 0.46	3.43 0.47	3.33 0.48

Overall students rate the perceived importance of learning, community, employability and health and well-being higher than their confidence in achieving them (see Table 6). Learning and health and well-being confidence were higher than community and employability confidence with community confidence ranking 4^th^ across 11 of the 12 disciplines of study. Learning importance was ranked highest in eleven of the twelve disciplines. Health and well-being importance was ranked lowest in 4 out the 12 disciplines of study. Computer science students reported the highest overall confidence score and Engineering, physics and mathematics students reporting the highest importance overall score. Healthcare and nursing sciences students reporting the lowest overall score for both confidence and importance. Individual discipline ranking per factor can be seen in Table 7.

**Table 6:** Domain and factor mean and ranking per discipline of study (LC = Learning Confidence; LI = Learning Importance; CoC = Community Confidence; CoI = Community Importance; EC = Employment Confidence; EI = Employment Importance; HC = Health and Wellbeing Confidence; HI = Health and Wellbeing Importance. Rank 1 = highest confidence or importance).

Discipline of Study	Confidence									Importance								
	LC	LC Rank	CoC	CoC Rank	EC	EC Rank	HC	HC Rank	Overall	LI	LI Rank	CoI	CoI Rank	EI	EI Rank	HI	HI Rank	Overall
**Business**	3.44	2	3.41	4	3.42	3	3.48	1	3.44	3.66	1	3.49	2	3.35	3	3.34	4	3.46
**Law**	3.44	3	3.16	4	4.43	1	3.47	2	3.37	3.58	1	3.34	3	3.33	4	3.42	2	3.42
**Architecture & Built Environment**	3.46	=2	3.38	4	3.46	=2	3.51	1	3.45	3.68	1	3.50	2	3.36	4	3.40	3	3.48
**Communities & Education**	3.31	3	3.05	4	3.35	2	3.37	1	3.27	3.63	1	3.26	3	3.17	4	3.38	2	3.35
**Computer Science**	3.48	3	3.45	4	3.55	1	3.54	2	3.51	3.71	1	3.57	2	3.41	3	3.39	4	3.52
**Design, Arts & Creative Industries**	3.42	1	3.09	4	3.33	3	3.35	2	3.30	3.66	1	3.45	2	3.30	3	3.26	4	3.42
**Engineering, Physics and Mathematics**	3.40	3	3.38	4	3.44	2	3.57	1	3.45	3.70	1	3.51	2	3.49	3	3.41	4	3.55
**Geography & Natural Sciences**	3.33	1	3.18	4	3.24	3	3.32	2	3.27	3.70	2	3.39	3	3.33	4	4.30	1	3.45
**Healthcare & Nursing Sciences**	3.03	3	2.71	4	3.19	2	3.28	1	3.05	3.43	1	2.62	4	2.96	3	3.39	2	3.08
**Humanities & Social Sciences**	3.48	1	3.05	4	3.29	3	3.43	2	3.31	3.73	1	3.20	4	3.31	3	3.46	2	3.42
**Psychology**	3.19	2	2.97	4	3.06	3	3.29	1	3.13	3.68	1	3.28	3	3.23	4	3.33	2	3.37
**Sport, Exercise & Rehabilitation**	3.33	4	3.37	3	3.46	2	3.58	1	3.44	3.63	1	3.57	2	3.29	4	3.38	3	3.45
**Overall**	3.42		3.32		3.42		3.47		3.41	3.67		3.45		3.33		3.37		3.45

**Table 7:** Discipline ranking per factor and domain (LC = Learning Confidence; LI = Learning Importance; CoC = Community Confidence; CoI = Community Importance; EC = Employment Confidence; EI = Employment Importance; HC = Health and Wellbeing Confidence; HI = Health and Wellbeing Importance. Rank – 1 = highest confidence or importance 12 = lowest confidence or importance).

	Confidence					Importance				
	LC	CoC	EC	HC	Overall	LI	CoI	EI	HI	Overall
**Business**	=4	2	6	5	=4	=7	5	4	10	4
**Law**	=4	7	1	6	6	11	8	=5	3	=7
**Architecture & Built Environment**	3	=3	=3	4	=2	=5	4	3	5	3
**Communities & Education**	10	=9	7	8	=9	=9	10	11	=8	10
**Computer Science**	=1	1	2	3	1	2	=1	2	=6	2
**Design, Arts & Creative Industries**	7	8	8	9	8	=7	6	8	12	=7
**Engineering, Physics and Mathematics**	6	=3	5	2	=2	=3	3	1	4	1
**Geography & Natural Sciences**	=8	6	10	10	=9	=3	7	=5	1	=5
**Healthcare & Nursing Sciences**	12	12	11	12	12	12	12	12	=6	12
**Humanities & Social Sciences**	=1	=9	9	7	7	1	11	7	2	=7
**Psychology**	11	11	12	11	11	=5	9	10	11	11
**Sport, Exercise & Rehabilitation**	=8	5	=3	1	=4	=9	=1	9	=8	=5

## Discussion

The present study examined pre-arrival confidence and importance ratings of PGT students across twelve academic disciplines at a UK HE institution, whilst considering the influence of gender, discipline of study, socioeconomic and international student status. The findings provide valuable insights into how postgraduate students’ pre-arrival expectations vary across disciplines and demographic groups as they prepare to start their PGT journey which has important practical implications.

### Pre-arrival domain differences

Consistent with previous findings in UG students [10], PGT students across all disciplines rated the perceived importance of learning, community, employability, and health and well-being higher than their confidence in achieving these domains.¹¹ Students recognising domains’ importance whilst doubting their preparedness may experience heightened anxiety. This might be particularly the case considering the compressed time frames in PGT requiring students to “hit the ground running”. However, this confidence-importance gap is narrower at PGT level compared to earlier findings in UG students [10]. This could indicate that PGT students have more realistic expectations calibrated by prior UG experience, or that they represent a self-selected group with enhanced confidence developed through successful degree completion and potential professional experience. These findings suggest that whilst PGT students arrive better calibrated than undergraduates, institutions should still provide intensive but brief transition support that validates students’ recognition of domains’ importance whilst building practical confidence through early successes and peer support.

Mirroring research in UG students [11], community confidence emerged as the lowest-ranked domain for PGT students, with community confidence ranking fourth (lowest) across eleven of the twelve schools. Many PGT programmes attract diverse cohorts in terms of age, cultural background, and prior experience, which may simultaneously enrich learning whilst complicating social integration [27,28]. The intensive academic demands of PGT study may also leave limited time for community engagement activities (such as sporting participation and institutional societies), particularly for students balancing study with work or family responsibilities [5]. The consequences of low community confidence extend beyond social satisfaction. Research in HE consistently demonstrates that sense of belonging and peer support networks contribute significantly to academic persistence, well-being and learning outcomes [29]. PGT students who struggle to develop community connections may be at elevated risk of isolation, particularly international students living away from home and immediate family or those unfamiliar with their institution [30]. Furthermore, professional networking opportunities, which constitute valuable outcomes of PGT study, depend substantially on social integration and community participation [31]. Universities should therefore prioritise community-building initiatives specifically designed for PGT cohorts that differ from UG fresher programmes given PGT students’ heterogeneity, compressed time frames and diverse commitments including work and family responsibilities [4,5].

Consistent with patterns in UG students [10,11], learning importance was ranked highest across eleven of twelve disciplines for PGT students. This universal recognition of learning’s importance reflects the fundamental academic purpose driving HE enrolment [32]. However, the nature of “learning” at PGT level differs qualitatively from UG study, requiring greater independent scholarship, critical evaluation, and original contribution to knowledge [33,34]. PGT students’ high importance ratings may not fully capture the shift in learning approaches required, suggesting potential for expectation-reality mismatches once programmes commence [35]. Institutions should provide explicit guidance regarding PG-level learning expectations, helping students understand not merely the importance of learning but the fundamentally different nature of PGT study.

Amongst PGT students, health and well-being importance ranked lowest in four of the twelve disciplines, whereas this pattern was not evident at UG level where health and well-being importance was consistently ranked second or third in eleven of the twelve schools [11]. This may indicate that mature PGT students possess established well-being strategies, or concerningly, that they anticipate sacrificing well-being for academic objectives, aligning with research documenting elevated PG stress and burnout [36]. Universities must ensure that well-being support is not only available but actively promoted and normalised within PGT cultures [37].

### Discipline of study differences

Disciplinary variations, whilst present, appear less pronounced at PGT than UGT level [10,11]. This may be due to a number of factors. Firstly, PGT applicants typically research programmes more thoroughly than UGT applicants [38] leading to more informed and realistic expectations aligned with programme characteristics. Secondly, prior HE experiences may provide PGT students with generalisable study skills and institutional navigation skills [34] that reduce discipline-specific variation. Thirdly, many PGT programmes explicitly select for students demonstrating preparedness through prior academic achievement and relevant experience, potentially screening out individuals with substantially lower confidence who might have entered UG study. These mechanisms collectively suggest that whilst disciplinary cultures remain important, their influence on pre-arrival expectations may be reduced at PGT level compared to UGT entry.

Computer science PGT students reported the highest overall confidence, this likely reflects the strong labour market demand and clear career pathways characterising the computing sector [39]. The technology industry’s well-publicised skills shortages and competitive graduate salaries may enhance computer science students’ confidence in employability domains. Research demonstrates that self-efficacy beliefs among computer science students are positively associated with deeper learning approaches and academic confidence [40]. This high confidence could be leveraged through peer mentoring schemes supporting students in lower-confidence disciplines. Healthcare and nursing sciences students reported lowest scores for both confidence and importance, contrasting sharply with their 3^rd^ and 4^th^ highest UG rankings. This may reflect differences between cohorts. Many PGT healthcare programmes attract mature career-changers possessing less domain-specific confidence than UG nursing students selecting healthcare as first careers [41]. Additionally, PGT programmes leading to advanced roles with substantial clinical responsibility may heighten perceived demands despite prior experience [42]. Institutions offering healthcare PGT programmes should investigate whether low pre-arrival scores translate into adjustment difficulties and consider enhanced transition support specifically for this cohort.

Overall, these disciplinary differences support longstanding evidence that disciplinary cultures can exert influence on students’ perceptions, priorities, and transition experiences [43]. Similarly, professional identity formation is likely to influence disciplinary differences with some disciplines associated with competitive labour markets and strong career orientation [44]. This might result in students prioritise employability and networking. It will be important for institutions to recognise these discipline differences for the design and implementation of transition support.

### Gender status

Male PGT students reported significantly higher community confidence and employability importance than females. Higher male community confidence may reflect greater confidence in professional networking contexts, driven by socialisation patterns encouraging assertiveness [27]. At PGT level, where cohorts are smaller and less structured, informal networking assumes greater importance, potentially advantaging male students in building valuable professional networks. Institutions should look to must develop inclusive environments that explicitly address professional networking inequalities such as through structured mentorship programmes that connect female PGT students with senior women in their fields or facilitated networking events designed to reduce gender-based confidence gaps.

Male students’ higher employability importance ratings may suggest more instrumental pursuit of PGT study prioritising career advancement. Female students may emphasise intrinsic learning and personal development, consistent with expectancy-value theory [28]. Expectancy-value theory [28] suggests that achievement-related decisions and persistence are shaped both by anticipated success and by the subjective value individuals assign to outcomes. Research shows women conceptualise success more broadly, emphasising work-life balance and meaningful work alongside traditional markers [29]. However, lower importance ratings do not indicate lower employability aspirations; rather, female students may conceptualise career success more holistically. Institutions should affirm diverse conceptualisations of career success that encompass work-life balance, meaningful contribution, and personal growth alongside traditional markers of professional achievement.

Unlike the UG study where female students rated learning, community, and health and well-being importance significantly higher than males [11], these differences were largely absent at PGT level. The merging of importance ratings by gender at PGT level suggests that mature reflection, life experience, or the professional orientation of PGT study may moderate some gender differences evident during adolescent and young adult development. Alternatively, the PGT cohort may represent a more selected subgroup where individuals of all genders have developed sophisticated understanding of HE’s multiple values.

### International status

International PGT students demonstrated significantly higher confidence and importance ratings across nearly all domains compared to domestic students. International students represent exceptionally self-selected populations characterised by high motivation, resilience, and self-efficacy, having navigated complex visa processes and substantial financial investments [45,46]. The decision to study internationally itself requires considerable confidence in one’s capacity to adapt to unfamiliar contexts. These characteristics may drive both elevated confidence (stemming from substantial prior achievement required to access international study) and elevated importance ratings (reflecting the career imperatives motivating international study).

However, substantially higher pre-arrival scores should not suggest reduced support needs. Extensive research documents challenges including language barriers, unfamiliar pedagogical practices, cultural adjustment, and social isolation [21,46]. Institutions must develop sophisticated approaches to international PGT student support that acknowledge these students’ considerable strengths and resilience whilst simultaneously providing robust scaffolding for cultural, linguistic, and professional transition. Support should commence pre-arrival, helping international students develop realistic expectations about challenges they will encounter whilst validating their existing competencies. Peer mentoring programmes connecting new international students with those who have successfully navigated similar transitions can provide valuable cultural navigation support. Employability provision must explicitly address international graduate employment pathways, providing realistic guidance about UK labour market access alongside support for home-country or global career development.

### Socio-economic status

The absence of significant socioeconomic effects at PGT level mirrors UG findings [11]. This suggests disadvantaged students successfully completing UG study and those progressing to PGT education represent particularly resilient subgroups overcoming structural barriers [26]. By PGT enrolment, these students may have developed comparable confidence through successfully navigating prior challenges aligning with research on “survivor bias” where individuals who persist despite adversity demonstrate characteristics distinct from the broader population [47]. However, this absence of differences should not suggest disadvantage no longer matters. Financial support, flexible delivery, and inclusive environments remain essential. Pre-arrival confidence may not capture full complexity. For example, disadvantaged PGT students may encounter greater challenges related to financial pressure, limited social capital, and restricted access to unpaid opportunities. Additionally, the socioeconomic measures of the current study may inadequately capture the multifaceted disadvantage at PGT level, for example current financial circumstances and caring responsibilities for mature students. Institutions must therefore maintain structural support addressing financial, social capital, and professional networking disadvantages that may emerge during programme participation.

### Limitations and future research

These findings must be interpreted considering the sample’s composition, where international students comprised 82% of respondents and the discipline of Business contributed 37% of the sample. The substantial international student overrepresentation (exceeding typical UK PGT proportions of approximately 43%) [18] means that the elevated confidence and importance scores observed overall likely reflect international students’ characteristics rather than representing all PGT students equally. Similarly, the concentration of business respondents suggests that average findings may be skewed toward perspectives characteristic of vocationally-oriented, career-focused programmes rather than representing the full diversity of PG taught study across disciplines.

The cross-sectional design captures expectations at a single pre-arrival timepoint, preventing examination of how expectations evolve during transition and how they relate to subsequent outcomes. Longitudinal research using the pre-arrival survey at regular timepoints throughout the PGT student journey from pre-arrival through to programme completion would establish whether initial expectations predict adjustment, well-being, and achievement. In addition, the study was conducted at a single UK institution, limiting generalisability. Multi-institutional research would clarify whether observed patterns reflect universal features of PGT transitions or institution-specific characteristics. Finally, the survey measures subjective confidence and importance ratings but does not assess actual competencies or objective preparedness. Future research might incorporate skills assessments alongside self-reported confidence to examine agreement. Qualitative research exploring meanings students attach to confidence and importance ratings would enrich understanding of cognitive and experiential foundations underlying survey responses. Further comparative research across UG, PGT, and postgraduate research cohorts would highlight how expectations shift across educational levels.

## Conclusion

This study examined pre-arrival confidence and importance ratings of PGT students across twelve academic disciplines at one UK HE institution, revealing both similarities with UG patterns and PGT specific characteristics. The findings demonstrate that discipline of study, gender, and international student status significantly influence expectations students bring to PGT study, whilst socioeconomic indicators show minimal effects at programme entry. The narrower confidence-importance gap compared to undergraduates suggests more calibrated expectations, but persistent gaps particularly in community domains indicate ongoing support needs. International students’ substantially elevated scores across all domains highlight both this group’s resilience and their potential vulnerability when optimistic expectations encounter challenging realities. These findings underscore the necessity for PGT transition support that acknowledges level-specific characteristics whilst remaining sensitive to disciplinary and demographic variations. The validated pre-arrival survey provides institutions with a robust tool for understanding incoming PGT populations and adapting teaching provision accordingly. As PGT education continues to evolve, understanding the varied expectations students bring to this critical educational transition will remain fundamental to ensuring meaningful engagement and successful outcomes across the increasingly diverse student population.
